# Carisoprodol Single and Multiple Dose PK-PD. Part II: Pharmacodynamics Evaluation Method for Central Muscle Relaxants. Double-Blind Placebo-Controlled Clinical Trial in Healthy Volunteers

**DOI:** 10.3390/jcm11041141

**Published:** 2022-02-21

**Authors:** Aitana Calvo, Mercedes González-Hidalgo, Ana Terleira, Nieves Fernández, Antonio Portolés

**Affiliations:** 1 Clinical Pharmacology Department, Hospital Clínico San Carlos, IdISSC, 28040 Madrid, Spain; aitanacalvo@hotmail.com (A.C.); anaisabel.terleira@salud.madrid.org (A.T.); 2 Medical Oncology Department, Hospital Universitario Gregorio Marañón, 28007 Madrid, Spain; 3 Clinical Neurophysiology Department, Hospital Clínico San Carlos, IdISSC, 28040 Madrid, Spain; mariamercedes.gonzalez@salud.madrid.org; 4 Belmac Laboratories, 28700 Madrid, Spain; nfernandez@faes.es; 5 Pharmacology & Toxicology Department, Facultad de Medicina, Universidad Complutense de Madrid, 28040 Madrid, Spain

**Keywords:** carisoprodol, meprobamate, dependence, pharmacodynamics, pharmacokinetics, sedation, psychomotor impairment, neuromuscular, central muscular relaxants

## Abstract

Centrally acting skeletal muscle relaxants (CMR) such as carisoprodol are used to treat acute, painful musculoskeletal conditions, though its precise mode of action has not been characterized. A double-blinded, placebo-controlled, randomized clinical trial was designed to evaluate the pharmacokinetics–pharmacodynamics (PKPD) of CMR after single (350 mg), double (700 mg), and multiple doses (up to 350 mg/8 h, 14 days) of carisoprodol. Muscular (Electromyogram–EMG, muscular strength dynamometry), central (sedation), and tolerability (psychomotor activity test, adverse events) parameters, as well as withdrawal symptoms, were evaluated. Thirteen healthy volunteers were enrolled. No evidence of direct muscle relaxation was evidenced, but some differences on sedation were evidenced throughout the study, suggesting that CMRs act, at least partly, through sedation. Most significant differences were detected at 1.5 h after dosing. The effect on psychomotor impairment was variable, most prominently after 1.5 h, too, suggesting that it is produced by carisoprodol rather than by meprobamate. No withdrawal symptoms were detected, so the risk of dependence following maximum doses and duration of treatment recommended, and under medical supervision, should be low.

## 1. Introduction

An effect of centrally acting skeletal muscle relaxants (CMR) through the blockage of synapses of the spinal cord in relevant animal models has been described, though in humans, this effect has not been shown, and an alternative hypothesis suggests that their effect is a result of sedation [1,2,3,4,5,6]. In fact, there has been no agreement on the correct methodology for a pharmacodynamic evaluation for CMR in the literature.

Carisoprodol is a CMR which was authorized in 1959 and is used in the US and Canada in combination with analgesics for lower back pain. In addition to the uncertainties regarding CMR in general, it is also unclear regarding carisoprodol, whether meprobamate, its main active metabolite, is responsible for carisoprodol’s effects or whether some of these can be attributed to the parent drug [7].

Reports on abuse of carisoprodol, usually in patients with a previous history of drug abuse, have been reported [4,8], usually in patients with long-term treatments (over 3 months) [1,5,8,9,10]. The typical withdrawal symptoms are similar to those of barbiturates, which include anxiety, tremor, insomnia, jittering, muscle twitching, and hallucinations. Symptoms have been reported 24 to 48 h after drug withdrawal and have reached its zenith 3 to 4 days later [10].

Therefore, the pharmacodynamic (PD) objectives of the study were to study parameters of central action by exploring somnolence as perceived by the investigator or the subject himself and to explore whether carisoprodol’s effect, as a model for other CMRs, could be studied by electromyography. An attempt to correlate the effects with carisoprodol’s or meprobamate’s concentrations in order to attribute the effects to parent drug or metabolite was also performed. Lastly, tolerability, both through adverse event (AE) collection (including withdrawal symptoms) and psychomotor impairment, was explored. The recommended conditions of use were followed. Additionally, the study was designed to estimate the pharmacokinetics of carisoprodol and meprobamate.

We consider that the study does not only address the safety problems of carisoprodol but might be of utility for other muscle relaxants whose mechanisms of action have not been characterized.

## 2. Materials and Methods

A randomized, crossover, 2-period design was used. The trial was double-blinded and controlled with placebo, as it was very important to limit unconscious bias both for investigators and for subjects as the PD endpoints explored, such as psychomotor impairment of somnolence, were considered extremely sensitive to identification of treatment. Treatment sequences were assigned according to a randomization list.

Key inclusion criteria included being of an age between 18 and 40 years, 12-lead electrocardiogram (ECG) within normal standards, a body mass index below or equal to 30 kg/m^2^, and normal hematology and biochemistry blood and urine analyses. A negative result for a quick test for beta-HCG was required, and an effective contraception method during the study was also compulsory for female subjects. Negative results on urine drug screen were required within 2 weeks before study participation. Key exclusion criteria included acute or chronic disease and regular use of medication. Further exclusion criteria were a personal history of hypersensitivity or photosensitivity to any drug; smoking, intention to donate blood, or to participate in another study for the following months; or any clinically relevant abnormalities, including vital signs. For inclusion criteria, please see part I [11].

Written informed consent was obtained by each subject following the principles of GCP (Good Clinical Practice). The trial was performed in the Clinical Pharmacology Studies Unit of the HCSC (Hospital Clinico San Carlos of Madrid, Spain) and was authorized by the Ethics Committee and the Spanish Agency on Medicines and Sanitary Products (IRB registry number: 06/230; EudraCT: 2006-004254-24). The principles of the Declaration of Helsinki and its subsequent revisions were observed.

Carisoprodol (350 mg tablets) or placebo was administered at increasing doses (every 12 h for 6 days and every 8 h thereafter for 6 additional days). After at least 14 days of washout, volunteers were crossed over to the alternate arm. Based on a different randomization list, on D7, a single 700 mg of carisoprodol or placebo was administered. Subjects received increasing doses in order to facilitate tolerability and avoid drop-outs.

Assignment of subjects to the treatment sequence was blindly designed by a computer-generated randomization table, balanced by blocks. Another randomization sequence was performed for the double dose (AA) or placebo (PP) at D7.

The study plan for most relevant activities is described in Table 1.

The pharmacokinetics (PK) of carisoprodol and meprobamate after single, multiple, and double doses was also studied. Samples were taken at baseline (+0.5; +1; +1.5; +2; +3; +4; +5; +6; +7; +8; +10; +12; +24), and PK parameters for both carisoprodol and meprobamate were calculated (AUC0-∞, AUC0-t, AUC0-8, AUC0-12, Cmax, and Tmax). Given the complexity and extension of all procedures, it has been published in two separate parts (I: PK; II: PD). For detailed results, please see part I [11].

### 2.1. PD Endpoints (Activity)

The following parameters on activity were assessed at baseline, +1.5 h and +5 h after drug administration, in the corresponding evaluation cross-over periods (active—carisoprodol—or placebo), D1 (single 350 mg dose), D14 (after 350 mg/12 h 6 days and 350 mg/8 h following 6 days), as well as at day 7 (D7) (randomly assigned placebo or carisoprodol 700 mg).

#### 2.1.1. Muscle Relaxation

##### Electromyogram

Electromyograms (EMG) were performed as described in Table 1. Disposable surface electrodes were attached to the participant’s forehead, and repetitive stimulation of the left facial nerve was undertaken. The recording electrode was placed on the frontalis muscle and the bipolar electrode on the mastoid. A motor evocated potential with supramaximal stimuli was estimated, and repetitive stimulation of the nerve was induced with a constant intensity and a 3 Hz frequency. The maximum amplitude of frontalis muscle action potential and the incidence of muscle fatigue were calculated.

##### Dynamometry

Measurements of bilateral handgrip strength using a hand dynamometer were performed. Training sessions were conducted. For each hand, the average of two readings was calculated. Grip strength is used at preclinical drug development to estimate activity [12,13,14]. Standard recommendations for performance were followed [15].

#### 2.1.2. Central Activity: Sleepiness Tests

In order to avoid a learning effect and the anxiety produced by psychomotor tests, training sessions were undertaken. The environment was the same during training and experimental sessions (same light intensity, space, workstation ergonomics, and similar noise and temperature). In addition, the tests were administered with the same instructions and in the same order. There were only two investigators administering tests, and they intentionally had the same attitude.

Perceived somnolence was determined by investigators and subjects using the SSS (Stanford Sleepiness Scale (SSS) and VAS (Visual Analogue Scale) of 100-mm [16,17,18,19,20,21].

### 2.2. Tolerability: Psychomotor Impairment, Withdrawal Symptoms, and Adverse Events

#### 2.2.1. Psychomotor Impairment

Tests were performed after sleepiness tests in a similar manner. A battery of psychomotor performance tests that have been used in clinical trials following the administration of different sedatives [17,18,19,20,21,22,23,24,25,26,27,28,29,30,31] were selected.

The original version of the Weschler Digit Symbol Substitution Test (DSST or Numbers Key) [32] and a modified version of the Cancellation Test (CT) were employed. The parameters that were recorded were the omitted cancellations, the time required to complete the test, the time—1 s per omission (DSST) and correct answers (CT).

Subjects were also asked to add up a sequence of 10 one-digit numbers, and the time required to perform the sums was recorded, assuming there would be no mistakes.

In addition, in order to estimate SVRT (simple visual reaction time), computers were displayed, and subjects were instructed to press the mouse when a blue rectangle appeared on screen. After 10 trials, the average latency time before response was measured.

#### 2.2.2. Adverse Events (AE)

Safety was monitored by AE recording throughout the study, together with vital signs, laboratory tests, and ECG at inclusion and after treatment

#### 2.2.3. Withdrawal Symptoms

Withdrawal symptoms were evaluated according to the American Psychiatric Association Diagnostic and Statistical Manual of Mental Disorders (DSM-IV) Diagnostic Criteria for Sedative, Hypnotic, or Anxiolytic Withdrawal on Days 1, 5, and 7 of each period.

### 2.3. Statistical Analysis

A minimum sample size of 12 subjects was calculated, as the crossover design allowed a small sample size and since the PD evaluation in the study was considered exploratory.

The descriptive statistics on PD parameters and differences versus baseline values were calculated (mean, standard deviation, standard error, median, maximum and minimum values). Graphs of the PD results vs. time were planned. To explore the effects of the drug and to investigate time vs. baseline changes, ANCOVA (model of Analysis of Covariance) and MANOVA (Multivariate analysis of variance) were performed for the dependent endpoints. PD measurements were considered dependent variables and used as quantitative endpoints, and the placebo and carisoprodol were considered independent factors. SPSS release 14 and WinNONLIN 4.1 were used.

## 3. Results

**Figure 1:**
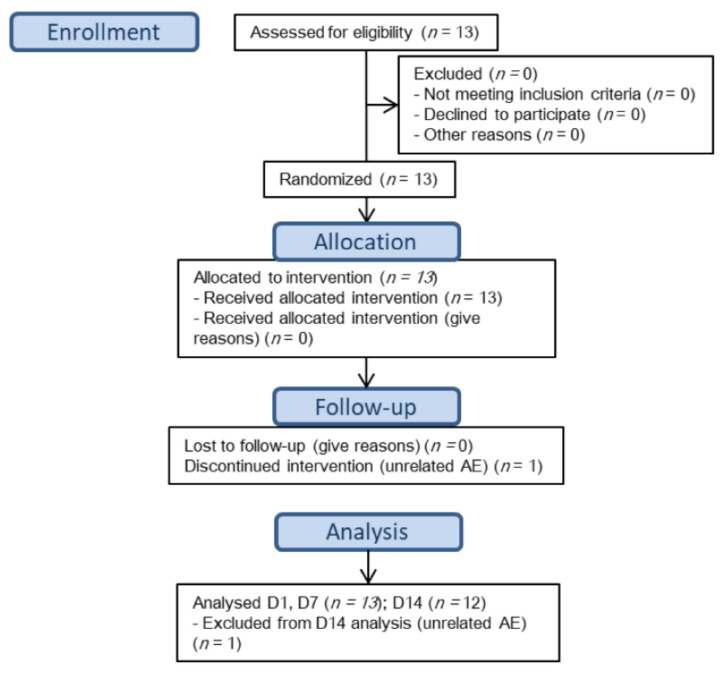
Subject Flow chart.

The subjects’ flow chart is shown in Figure 1. For PK results and demographics, please see part I [11]. For descriptive statistics of PD parameters please see Appendix A (Table A1, Table A2, Table A3, Table A4 and Table A5).

### 3.1. Muscle relaxation

#### 3.1.1. Electromyogram (EMG)

Baseline values were similar for both groups. There were no statistically significant differences with the MANOVA or ANCOVA in the amplitude of the action potential. However, there was a trend in the appearance of fatigue of the action potential after repetitive stimuli at +5 h after a 700 mg dose of carisoprodol (MANOVA) (*p* = 0.092).

#### 3.1.2. Dynamometry

**Figure 2:**
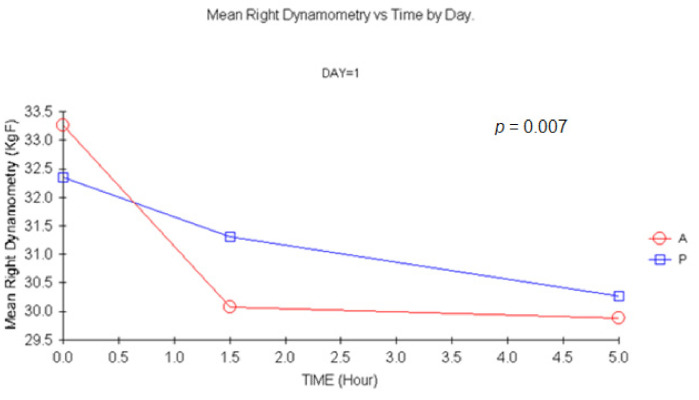
Mean Right hand Dynamometry vs. time at D1. (1.5 h vs. baseline: *p =* 0.007, MANOVA). A: carisoprodol 350 mg; P: placebo.

**Figure 3:**
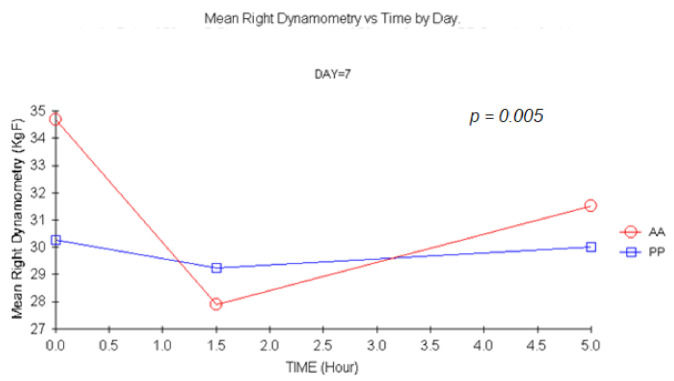
Mean Right hand Dynamometry vs. time at D7. (1.5 h vs. baseline: *p* = 0.005, MANOVA). AA: Carisoprodol 350 mg 2 tablets; PP: placebo 2 tablets.

**Figure 4:**
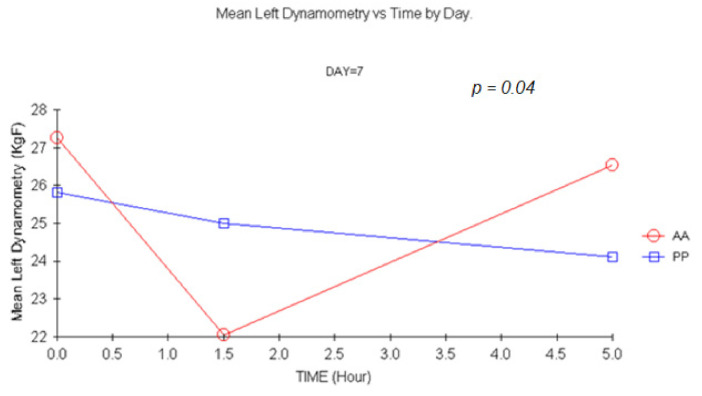
Mean Left hand Dynamometry vs. time at D7. (1.5 h vs. baseline: MANOVA *p =* 0.004; ANCOVA *p* = 0.0044). AA: Carisoprodol 350 mg 2 tablets; PP: placebo 2 tablets.

There were statistically significant changes for carisoprodol vs. placebo +1.5 h for single and double doses (*p =* 0.007 and *p =* 0.005, respectively) for MANOVA. Similar results were shown for left-hand dynamometry, though statistically significant changes were only detected 1.5 h after a double dose (*p =* 0.044) upon ANCOVA. This result was confirmed with MANOVA (*p =* 0.04). The more representative profiles are shown in Figure 2, Figure 3 and Figure 4.

### 3.2. Central Activity: Sleepiness Tests

#### 3.2.1. Subject’s and Investigator’s Perceived Somnolence VAS

**Figure 5:**
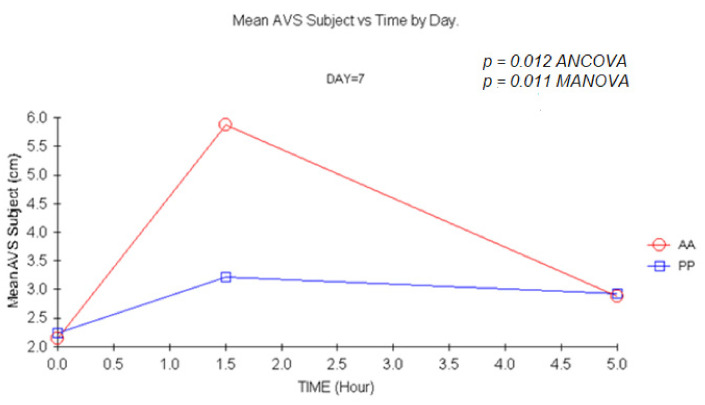
Mean VAS subject vs. time at D7. (1.5 h vs. baseline: *p =* 0.012, ANCOVA and *p =* 0.011, MANOVA). AA: Carisoprodol 350 mg 2 tablets; PP: placebo 2 tablets.

**Figure 6:**
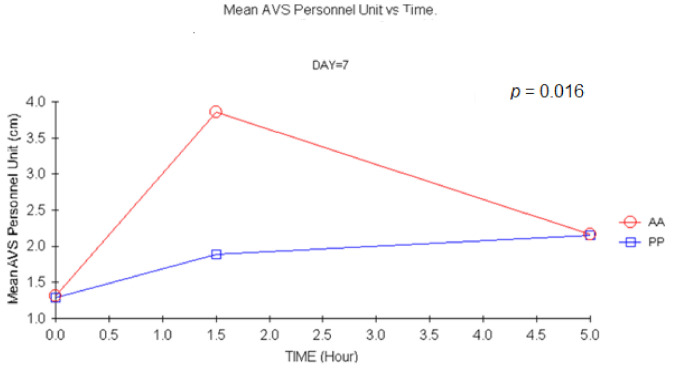
Mean investigator’s perceived VAS vs. time at D7 (1.5 h vs. baseline: *p =* 0.006, ANCOVA). AA: Carisoprodol 350 mg 2 tablets; PP: placebo 2 tablets.

There was a trend for an increase in somnolence as assessed by the subjects (Figure 5 and Figure 6) of the group receiving carisoprodol 1.5 h after single and multiple doses (*p =* 0.06 1.5 h post-dose on day 1; *p =* 0.07 1.5 h post-dose on day 14) with ANCOVA, which were statistically significant upon MANOVA, *p =* 0.03 and *p =* 0.05, respectively). An increase of a bigger magnitude was evidenced after double doses (*p =* 0.012 and *p =* 0.011, upon ANCOVA and MANOVA, respectively).

An increase of a larger magnitude in the degree of somnolence as per subject’s VAS was evidenced after a 700 mg dose, reaching 4.3 cm on a 10 cm scale at 1.5 h. Placebo effect was higher after a double dose (ANCOVA; *p =* 0.012).

Statistically significant differences for carisoprodol versus placebo on VAS as per the investigators were evidenced after a double dose at 1.5 h (*p =* 0.006) (Figure 6) and after multiple doses (*p =* 0.025).

#### 3.2.2. Subject’s and Investigator’s Stanford Sleepiness Scale (SSS)

Statistically significant differences as per subject’s SSS were detected with ANCOVA at 1.5 h on D1 (*p =* 0.05), D14 (*p =* 0.008), and D7 7 (*p =* 0.006).

**Figure 7:**
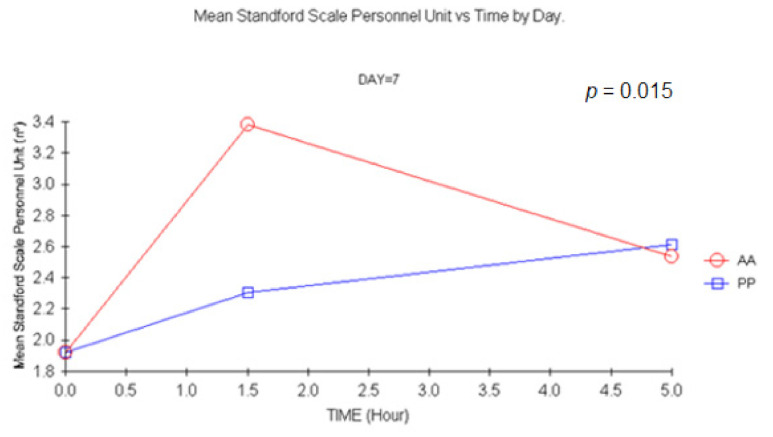
Mean Stanford scale by unit’s personnel vs. time at D7. (1.5 h vs. baseline: *p =* 0.015, ANCOVA). AA: Carisoprodol 350 mg 2 tablets; PP: placebo 2 tablets.

After multiple doses of treatment (D14), significant results were evidenced for 350 mg doses at 1.5 h (*p =* 0.017) and after a double dose at 1.5 h (*p =* 0.015) (ANCOVA) for the investigator (Figure 7).

## 4. Tolerability

### 4.1. Psychomotor Impairment

#### 4.1.1. Cancellation Test (CT)

There was a marked improvement in the subject’s score (decrease in the time to perform the task) from D1 to D14, reflecting a learning effect, despite training sessions.

**Figure 8:**
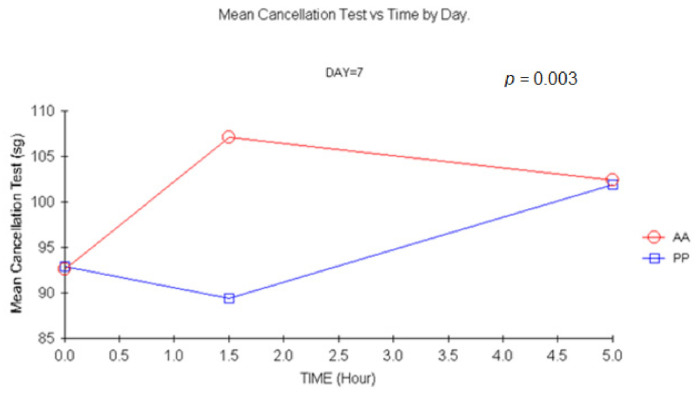
Mean Cancellation test vs. time at D7. (1.5 h vs. baseline: *p*: 0.003, ANCOVA). AA: Carisoprodol 350 mg 2 tablets; PP: placebo 2 tablets.

However, an increase in the time to perform the task was noted after a double dose. Changes were statistically significant +1.5 h on D14 (*p =* 0.03) and D7 (*p =* 0.003) with ANCOVA. Similar results were obtained with MANOVA (*p =* 0.048 and 0.02, respectively) (see Figure 8).

##### 4.1.2. Digit Symbols Substitution Test (DSST or Numbers Key Test)

**Figure 9:**
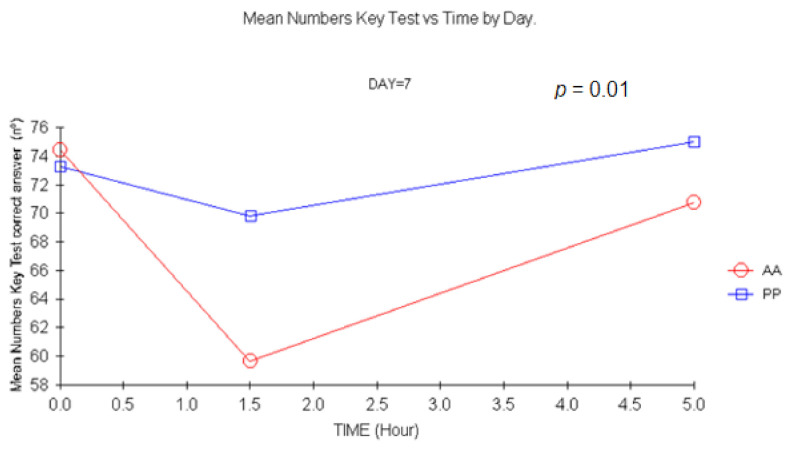
Mean DSST (Numbers key) test vs. time at D7). (1.5 h vs. baseline: *p =* 0.01, ANCOVA). AA: Carisoprodol 350 mg 2 tablets; PP: placebo 2 tablets.

The baseline values were similar for both groups for DSST. A decrease in the number of correct answers was evidenced +1.5 h after single, multiple, and double doses. Differences at +1.5 were statistically significant on D1 and D14 (*p =* 0.049, *p =* 0.09, respectively). A remarkable effect was evidenced after a double dose (Figure 9), as the number of correct answers decreased up to 16.5 (mean) (*p =* 0.01, ANCOVA).

##### 4.1.3. SVRT

**Figure 10:**
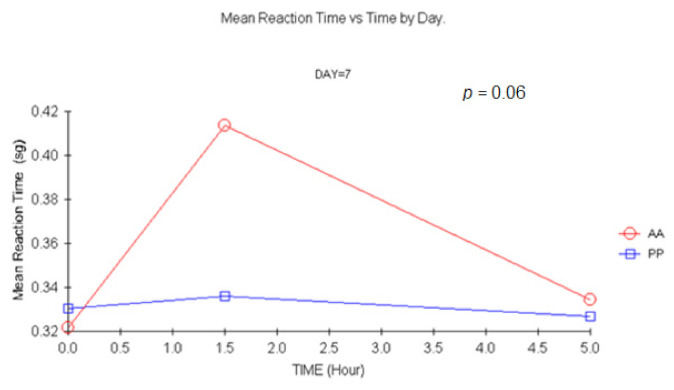
Mean Reaction time vs. time at D7. (1.5 h vs. baseline: *p =* 0.006, ANCOVA). AA: Carisoprodol 350 mg 2 tablets; PP: placebo 2 tablets.

There were small differences after single and multiple doses in the SVRT (Figure 10), although, for subjects that received a double dose, an increase of 0.1 s (in about 0.32 s) in the 1.5 h vs. baseline difference was detected. A tendency at 1.5 h on D1 (*p =* 0.06) was identified for subjects receiving carisoprodol. After double doses, there were statistically significant differences (*p =* 0.006, ANCOVA). There was a tendency for statistical significance on the number of mistakes on D7, 1.5 h, MANOVA (*p =* 0.054).

### 4.2. Adverse Events

All patients (100%) experienced at least one AE (regardless of causality). There were 58 AEs recorded, 28 of them after carisoprodol (48,3%). Moreover, 31 AEs had at least a possible causal relationship (53,4%), 9 occurred with placebo (15,5%) (4 headaches, 2 events of somnolence, 1 abdominal pain, 1 diarrhea, 1 erythema), and 22 with carisoprodol (37,9%) (13 somnolence, 3 headache, 2 transient anxiety, 1 insomnia, 1 dizziness, 1 hiccups, 1 nausea). There were no serious AEs, and all AEs were self-limited. The most frequent AE was somnolence.

### 4.3. Abstinence Criteria—Withdrawal Symptoms

No subject complied with DSM-IV withdrawal criteria, though five symptoms were registered (one event of nausea/vomiting, one insomnia, and two anxieties after active treatment; one anxiety event after placebo treatment).

## 5. Discussion

Given the safety concerns regarding abuse potential of carisoprodol [1,4,5,8,10,33,34,35,36,37] and the lack of precise knowledge regarding the mode of action of carisoprodol, or even whether it has an effect on itself, several PD parameters were chosen to estimate the action, tolerability, and ability to produce withdrawal symptoms of carisoprodol and its main active metabolite, meprobamate. In addition, since there is no agreement in the literature as to what methods would be most adequate to evaluate the action of CMR, central action parameters, such as sedation, as well as muscular action parameters, were explored. Since carisoprodol is not the only CMR whose mechanism of action is not known, a proposal for evaluating the effect of similar drugs with non-invasive methods is of the highest interest.

One of the main objectives of the trial was to investigate if the effect of CMR could be estimated by electromyography by measuring the incidence of fatigue and the action potential of the frontalis muscle. Similar techniques have been described in the literature for afloqualone, a completely different CMR [23,24], or for other substances, such as methocarbamol, propofol, etomidate, or midazolam [38,39].

No decrease in the amplitude of the action potential was evidenced after single, multiple, or double doses; however, a trend towards the appearance of fatigue of the action potential after repetitive stimuli at +5 h was evidenced after a 700 mg of carisoprodol (MANOVA) (*p =* 0.092).

Grip strength is frequently used in preclinical drug development to estimate toxicity but also to identify muscle relaxation in species such as mice [12,13,14,40,41,42]. It has also been used to determine muscular function and strength after curarizing agents during anesthesia [43]. Differences between subjects receiving carisoprodol or placebo were detected 1.5 h after treatment in the maximum strength exerted on day 1 and 7 for the right hand and on day 7 for the left hand. The decrease for both groups throughout the day could be probably attributed to a decrease in motivation.

In summary, no sign of direct muscle relaxation was present in our experiment, though a trend towards the appearance of fatigue of the action potential after repetitive stimuli at +5 h was evidenced after a double carisoprodol dose. However, given the small sample size, and due to the absence of validation of the technique, further tests should be performed before discarding employing EMG parameters for CMR. In contrast, to our knowledge it is the first time that grip strength has been used in humans to infer muscle relaxation. Unfortunately, this technique, and in opposition to what happens at non-clinical level, appears to be extremely sensitive to subject’s motivation. Statistically significant changes were shown at 1.5 h after single and double doses. As grip strength has also been employed as surrogate for neurotoxicity [14,43,44,45], this technique is of particular interest and should be further explored for other CMRs.

In addition, as these drugs are thought to exercise their action by means of sedation [16,17,18,19,20,21], we attempted to explore the PD of carisoprodol by measuring perceived somnolence, which appeared to have been short-lasting and related to the theoretical Cmax of carisoprodol (1.5 h) and not to that of meprobamate (5 h). In this study, PK for both meprobamate and carisoprodol were adequately characterized (please see part I), [11]. The Tmax was 1.19 (SD 0.69) and 3,77 (SD 1.47) for carisoprodol and meprobamate, respectively [11]. These data suggest that carisoprodol has an effect in itself, rather than being exclusively mediated through meprobamate.

Continuous VAS appears to have been slightly more sensitive to changes in somnolence than the discrete SSS, though both scales were capable of detecting significant differences. In addition, scales that rely on subject’s perception of somnolence were slightly more sensitive, probably confirming the need to incorporate subjective measurements into clinical trials.

The selected psychomotor performance tests have been used in clinical trials after the administration of different sedatives [17,22,23,24,25,26,27,28,29,30,31,32]. A learning effect could not be completely avoided, which is probably one of the main limitations of our study. Overall, the effect of carisoprodol on validated scales measuring psychomotor impairment was variable, with most scales detecting a limited effect at 1.5 h, mainly for the double dose. Again, the data confirm an effect of carisoprodol rather than being mediated by meprobamate. Given the effect on VRT, on DSST, and on CT, a clinically relevant effect in performing real-life complex activities cannot be excluded. Of note, it is reassuring that even in the absence of significant impairing effects, subjects did not underestimate their degree of somnolence. A limitation of this study could be that, as for many drugs, great interindividual variation, due to a certain extent to blood concentrations, could obscure some of the findings.

It could be questioned whether the doses utilized were too low to allow for a difference to be detected. However, the inclusion of 700 mg dosing makes this hypothesis unlikely, as it is the maximum dose under recommended conditions of use and sufficient to detect changes in psychomotor tests. It should also be noted that no elderly patients were included, and it is in these patients in whom the appearance of psychomotor impairment and sedation is more likely. On the other hand, selected subjects were young and probably not familiar with the general effects of CNS depressants.

There was no evidence of withdrawal symptoms after recommended conditions of use, as no subject complied with DSM-IV criteria. These data are concordant with published case reports for abuse, which suggest that dependence appears after consumption of carisoprodol ranging from 3 months to one year, usually in patients with a history of drug abuse [1,5,8,9,10]. It should be noted that the most frequent strategies to assess abuse liability of a drug include measuring subjective effects such as drug-liking, euphoria, and elation and objective effects such as psychomotor impairment. As mentioned above, the impairing effect of carisoprodol was lower than expected, though it was out of the scope of this trial to evaluate the abuse liability of carisoprodol. It should be noted, too, that the controlled conditions of a clinical trial are not representative of those situations in which conducts of dependence are prone to occur. However, the former can be comparable to a clinical situation with a strict medical supervision, and in this situation, together with a limited impairing effect and the absence of physical dependence under recommended conditions of use, the risk of dependence is probably low.

## 6. Conclusions

Tests for sedation and psychomotor impairment were sensitive in evaluating carisoprodol’s in accordance with previous hypotheses proposing that CMRs act, at least partly, through sedation. Most significant differences were detected 1.5 h after treatment, suggesting that carisoprodol has a centrally acting activity which is not mediated by meprobamate. No firm conclusion apart from a tendency to fatigue in the action potential can be drawn from electromyogram analyses, though some signals were detected, and these tests should be further explored in larger sample sizes.

Taken together, these findings emphasize the need to limit the use of the highest doses of carisoprodol. There was no evidence of withdrawal symptoms in our study, and the risk of dependence under recommended conditions of use and under strict medical supervision appears to be limited. Nonetheless, clinicians should be aware of the abuse potential of carisoprodol after long-term treatments.

## Figures and Tables

**Table 1:** Study Plan.

	Period A (Carisoprodol/Placebo)				Period B (Placebo/Carisoprodol)			
	D1	D7	D14	Day 14 Period A +48, 120 and 168 h	D1	D7	D14	Day 14 Period B +168–24 h
PK	✓	✓	✓		✓	✓	✓	
EMG	✓		✓		✓	✓	✓	
Sedation scales	✓	✓	✓		✓	✓	✓	
Psychomotor activity	✓	✓	✓		✓	✓	✓	
Dynamometry	✓	✓	✓		✓	✓	✓	
Adverse events	✓	✓	✓	✓	✓	✓	✓	✓
Withdrawal symptoms				✓				✓

PK: pharmacokinetics; EMG: electromyogram; Baseline, +1.5 h, +5 h.

## Data Availability

The data presented in this study are available on request from the corresponding author.

## Appendix A

**Table A1:** Descriptive statistics on the differences vs. baseline values of EMG fatigue after single (day 1), multiple (day 14) or double doses (day 7) or carisoprodol or placebo.

Drug Treatment Period		Fatigue (Dif %) Day 1 Difference Time 1.5 vs. 0	Fatigue (Dif %) Day 1 Difference Time 5 vs. 0	Fatigue (Dif %) Day 14 Difference Time 1.5 vs. 0	Fatigue (Dif %) Day 14 Difference Time 5 vs. 0	Fatigue (Dif %) Day 7 Difference Time 1.5 vs. 0	Fatigue (Dif %) Day 7 Difference Time 5 vs. 0
P	N	13	13	6	6	7	7
	Mean	−7.6956	−10.5613	0.8594	0.3353	−1.3249	2.1674
	SD	15.48327	14.85825	3.27662	3.85995	9.21977	6.46668
	Median	−5.0132	−4.9771	1.9944	0.8764	−0.2614	1.4006
A	N	13	13	6	6	6	6
	Mean	−5.1272	−7.0049	1.1844	−1.3189	3.7781	−4.3638
	SD	14.53944	14.73740	4.36398	5.00332	7.80195	3.10312
	Median	−6.5292	−4.0153	1.7131	−1.1970	.3377	−4.9519

**Table A2:** Descriptive statistics on the differences vs. baseline values of left- and right-hand dynamometry after single (day 1), multiple (day 14), or double doses (day 7) or carisoprodol or placebo.

Drug Treatment Period		Dynamometry Left Day 1 Difference 1.5 vs. 0	Dynamometry Left Day 1 Difference 5 vs. 0	Dynamometry Left Day 14 Difference 1.5 vs. 0	Dynamometry Left Day 14 Difference 5 vs. 0	Dynamometry Left Day 7 Difference 1.5 vs. 0	Dynamometry Left Day 7 Difference 5 vs. 0
P	N	13	13	12	12	13	13
	Mean	−1.8846	−2.2308	−0.5833	1.3750	−2.8077	−1.8077
	SD	4.37871	3.94554	2.89069	4.64232	5.13784	4.61221
	Median	−1.0000	−2.0000	0.0000	0.7500	−2.0000	− 2.0000
A	N	13	13	12	12	13	13
	Mean	−2.7308	−1.6538	−1.6667	−0.9583	−6.8077	−1.8077
	SD	5.95334	5.89980	5.34846	2.62383	5.47547	4.61221
	Median	−2.5000	−2.0000	−0.7500	−0.2500	−7.5000	−2.0000
**Drug Treatment Period**		**Dynamometry Right Day 1 Difference 1.5 vs. 0**	**Dynamometry Right Day 1 Difference 5 vs. 0**	**Dynamometry Right Day 14 Difference 1.5 vs. 0**	**Dynamometry Right Day 14 Difference 5 vs. 0**	**Dynamometry Right Day 7 Difference 1.5 vs. 0**	**Dynamometry Right Day 7 Difference 5 vs. 0**
P	N	13	13	12	12	13	13
	Mean	−1.0385	−2.0769	−0.1538	1.3462	− 3.2308	−1.3077
	SD	4.1052	5.5896	3.8643	4.7626	6.0883	3.4250
	Median	0.0000	−1.0000	−1.0000	1.0000	−2.0000	− 2.0000
A	N	13	13	12	12	13	13
	Mean	−3.1923	−3.3846	−3.2917	−0.0417	−4.6154	−2.1538
	SD	5.78986	5.45112	8.5718	3.81062	4.49608	4.41298
	Median	−4.5000	−3.0000	−1.0000	−0.5000	−2.0000	−0.5000

**Table A3:** Descriptive statistics on the differences vs. baseline values of subject’s perceived somnolence as per the VAS after single (day 1), multiple (day 14), or double (day 7) doses of carisoprodol or placebo.

Drug Treatment Period		VAS (cm) Day 1 Difference 1.5 vs. 0	VAS (cm) Day 1 Difference 5 vs. 0	VAS (cm) Day 14 Difference 1.5 vs. 0	VAS (cm) Day 14 Difference 5 vs. 0	VAS (cm) Day 7 Difference 1.5 vs. 0	VAS (cm) Day 7 Difference 5 vs. 0
Subject’s perceived somnolence							
P	N	13	13	13	13	13	13
	Mean	−0.2692	−0.4308	−0.6538	−0.8077	0.9769	0.6923
	SD	1.21955	2.46218	1.00715	1.75141	1.97448	2.69736
	Median	0.0000	0.0000	−0.8000	−1.1000	0.2000	0.3000
A	N	13	13	12	12	13	13
	Mean	0.9615	0.2846	0.3833	−0.6000	3.7308	0.7385
	SD	1.59767	1.60200	1.54027	1.29123	2.57403	2.16662
	Median	0.1000	0.0000	0.6500	−0.2500	4.3000	0.4000
Investigator’s perceived somnolence							
P	N	13	13	13	13	13	13
	Mean	−0.4769	0.0385	−0.7154	−0.6231	0.6077	0.8692
	SD	1.17200	1.43848	1.65068	1.80192	0.81595	1.08119
	Median	−0.6000	0.3000	−0.4000	−0.4000	0.7000	0.9000
A	N	13	13	12	12	13	13
	Mean	−0.1000	0.1615	0.2500	−0.8417	2.5462	0.8538
	SD	1.53025	1.96661	0.95964	0.98761	1.84915	1.85590
	Median	−0.3000	−0.2000	0.0500	−1.0500	2.1000	0.4000

**Table A4:** Descriptive statistics on the differences vs. baseline values of subject’s perceived somnolence as per the Stanford Sleepiness Scale after single (day 1), multiple (day 14), or double doses (day 7) of carisoprodol or placebo.

Drug Treatment Period		Stanford Scale Day 1 Difference Time 1.5 vs. 0	Stanford Scale Day 1 Difference Time 5 vs. 0	Stanford Scale Day 14 Difference Time 1.5 vs. 0	Stanford Scale Day 14 Difference Time 5 vs. 0	Stanford Scale Day 7 Difference Time 1.5 vs. 0	Stanford Scale Day 7 Difference Time 5 vs. 0
Subject’s Stanford Sleepiness Scale							
P	N	13	13	13	13	13	13
	Mean	−0.3846	−0.2308	−0.5385	−0.4615	0.6923	0.6923
	SD	0.76795	1.23517	0.87706	1.45002	1.10940	1.65250
	Median	0.0000	0.0000	−1.0000	−1.0000	0.0000	1.0000
A	N	13	13	12	12	13	13
	Mean	0.3846	0.0000	0.4167	−0.2500	2.4615	0.3077
	SD	0.96077	0.70711	0.90034	0.75378	1.61325	1.31559
	Median	0.0000	0.0000	1.0000	0.0000	3.0000	0.0000
Investigator’s Stanford Sleepiness Scale							
P	N	13	13	13	13	13	13
	Mean	−0.4615	−0.2308	−0.5385	−0.4615	0.3846	0.6923
	SD	0.87706	0.83205	0.87706	1.45002	0.86972	1.10940
	Median	−1.0000	0.0000	−1.0000	−1.0000	0.0000	1.0000
A	N	13	13	12	12	13	13
	Mean	−0.1538	0.0000	0.4167	−0.2500	1.4615	0.6154
	SD	0.98710	1.22474	0.90034	0.75378	1.05003	1.55662
	Median	0.0000	0.0000	1.0000	0.0000	1.0000	0.0000

**Table A5:** Descriptive statistics on the differences vs. baseline values of subject’s score on the psychomotor tests Cancellation test after single (day 1), multiple (day 14) or double doses (day 7) of carisoprodol or placebo.

Drug Treatment Period		Day 1 Difference 1.5 vs. 0	Day 1 Difference 5 vs. 0	Day 14 Difference 1.5 vs. 0	Day 14 Difference 5 vs. 0	Day 7 Difference 1.5 vs. 0	Day 7 Difference 5 vs. 0
Cancellation Test							
P	N	13	13	13	13	13	13
	Mean	−5.3077	−14.7692	−8.6923	−15.5385	−3.4615	9.0769
	SD	7.37546	8.44742	8.99430	11.55145	5.48658	11.47070
	Median	−5.0000	−15.0000	−7.0000	−16.0000	−4.0000	7.0000
A	N	13	13	12	12	13	13
	Mean	−1.8462	−10.6923	−2.5000	−12.2500	14.4615	9.7692
	SD	9.08154	7.26160	5.16104	10.04648	16.57114	17.27789
	Median	−3.0000	−12.0000	−3.0000	−12.0000	14.0000	7.0000
Digit Symbol Substitution Test (Numbers Key)							
P	N	13	13	13	13	13	13
	Mean	2.6923	1.9167	.3077	7.6667	−3.4615	1.6923
	SD	5.29756	5.66422	4.17102	6.59660	5.54700	4.66163
	Median	4.0000	2.5000	1.0000	10.5000	−2.0000	3.0000
A	N	13	13	12	12	13	13
	Mean	−1.6154	3.6923	−2.8333	6.6667	−14.8462	−3.6923
	SD	4.97558	3.17240	5.55687	6.51339	7.89352	12.65164
	Median	−3.0000	2.0000	−2.0000	7.0000	−12.0000	−1.0000
Simple visual reaction time (SVRT)							
P	N	13	13	13	13	13	13
	Mean	−0.012892	−0.018185	0.007608	0.010754	0.0058	−0.0034
	SD	0.0230643	0.0238022	0.0366391	0.0413096	0.05896	0.03816
	Median	−0.007000	−0.019100	0.005900	0.000000	−0.0029	0.0008
A	N	13	13	12	12	13	13
	Mean	0.011115	0.008192	0.008883	0.016058	0.0921	0.0128
	SD	0.0543668	0.0379932	0.0294313	0.0837221	0.07610	0.04407
	Median	−0.001000	−0.009500	−0.000900	−0.006750	0.0530	−0.0025
